# A Fast and Precise Indoor Localization Algorithm Based on an Online Sequential Extreme Learning Machine ^†^

**DOI:** 10.3390/s150101804

**Published:** 2015-01-15

**Authors:** Han Zou, Xiaoxuan Lu, Hao Jiang, Lihua Xie

**Affiliations:** School of Electrical and Electronics Engineering, Nanyang Technological University, 50 Nanyang Ave, Singapore 639798, Singapore; E-Mails: xlu010@e.ntu.edu.sg (X.L.); jiangh@ntu.edu.sg (H.J.); elhxie@ntu.edu.sg (L.X.)

**Keywords:** indoor localization, online sequential extreme learning machine, WiFi

## Abstract

Nowadays, developing indoor positioning systems (IPSs) has become an attractive research topic due to the increasing demands on location-based service (LBS) in indoor environments. WiFi technology has been studied and explored to provide indoor positioning service for years in view of the wide deployment and availability of existing WiFi infrastructures in indoor environments. A large body of WiFi-based IPSs adopt fingerprinting approaches for localization. However, these IPSs suffer from two major problems: the intensive costs of manpower and time for offline site survey and the inflexibility to environmental dynamics. In this paper, we propose an indoor localization algorithm based on an online sequential extreme learning machine (OS-ELM) to address the above problems accordingly. The fast learning speed of OS-ELM can reduce the time and manpower costs for the offline site survey. Meanwhile, its online sequential learning ability enables the proposed localization algorithm to adapt in a timely manner to environmental dynamics. Experiments under specific environmental changes, such as variations of occupancy distribution and events of opening or closing of doors, are conducted to evaluate the performance of OS-ELM. The simulation and experimental results show that the proposed localization algorithm can provide higher localization accuracy than traditional approaches, due to its fast adaptation to various environmental dynamics.

## 1. Introduction

The widespread usage of mobile devices and the popularity of social networks have spurred extensive demands on location-based service (LBS) in recent years. In outdoor environments, GPS has dominated the LBS market with excellent localization performance. However, due to the lack of line of sight (LoS) transmission channels between satellites and a receiver, GPS is not capable of providing positioning service with sufficient localization accuracy in indoor environments [1]. Hence, developing an indoor positioning system (IPS) to provide reliable and precise indoor positioning and navigation has become a hot research topic recently. Various wireless communication technologies have been studied and developed to provide indoor positioning and navigation services in the past two decades [2–5]. Unlike other wireless technologies, such as ultra-wideband (UWB) and radio frequency identification (RFID), which require the deployment of extra infrastructure, the existing IEEE 802.11 (WiFi) network infrastructures, such as WiFi routers, are widely available in large numbers of commercial and residential buildings, and nearly every mobile device now is equipped with a WiFi receiver. As such, it is low-cost and practical to develop a WiFi-based IPS to provide LBS in an indoor environment [6–11].

Fingerprinting-based localization algorithms are widely adopted for WiFi-based indoor positioning, since the received signal strengths (RSS) can be measured easily from mobile devices [7–12]. However, the existing WiFi-based IPSs adopting the fingerprinting approach suffer from two major problems. One is that the site survey involves intensive costs of both time and manpower during the offline calibration phase. In order to achieve sufficient localization accuracy, the WiFi RSS fingerprints from different access points (APs) need to be measured at a huge number of calibration points. The other one is that the existing fingerprinting-based approaches are not robust to environmental dynamics [6,13]. Since the WiFi RSS fingerprint database is built up during the offline phase, it cannot reflect the real-time radio map of the WiFi signals well once the environment is altered during the online localization phase. Environmental factors, such as presence of humans, opening and closing of doors and variations of humidity, can interfere with the propagation of WiFi signals severely [6]. This will lead to serious localization errors in the estimation of the target if the same WiFi RSS fingerprint database is adopted.

In this paper, we propose an indoor localization algorithm based on an online sequential extreme learning machine (OS-ELM) to address the above two problems accordingly. Originating from the batch learning extreme learning machine (ELM), OS-ELM inherits the advantage of ELM, which can provide good generalization performance at an extremely fast learning speed [14]. In addition, OS-ELM has an online sequential learning ability that does not require retraining when new data are received [15]. Another noteworthy point of OS-ELM is that, different from other online sequential learning algorithms, such as stochastic gradient descent back-propagation (SGBP) [16] and growing and pruning RBF network (GAP-RBF) [17], which require specific types of hidden nodes and can only handle data one-by-one (learning only one training sample at each time), OS-ELM is able to adapt to various types of hidden nodes and can learn data one-by-one or chunk-by-chunk with a varying chunk size. Therefore, WiFi RSS fingerprints can be collected and updated more flexibly for OS-ELM online sequential learning. In addition, the fast learning speed of OS-ELM greatly reduces time consumption and manpower costs for the offline site survey. More importantly, the online sequential learning ability of OS-ELM permits the entire system to provide sufficient localization accuracy, even under environmental dynamics. A preliminary version of this paper was presented at the 2014 IEEE World Forum on the Internet of Things (WF-IoT) Conference [18]. In addition to the results in [18], a more elaborate description of the OS-ELM approach is presented in this paper. Furthermore, a simulation is conducted and demonstrated, as well. Besides overall performance evaluation and comparison between OS-ELM and existing fingerprinting-based approaches, experiments under specific environmental changes, such as variations of occupancy distribution and events of opening or closing of doors, are also conducted to evaluate the performance of OS-ELM in this paper.

The rest of the paper is organized as follows. In Section 2, we review related works. Section 3 provides the proposed localization algorithm. The simulation results and evaluation of OS-ELM are presented in Section 4. In Section 5, a system overview of our WiFi-based IPS is provided firstly, followed by the experimental results and performance evaluation of the proposed localization algorithm. We conclude the work in Section 6.

## 2. Related Works

Indoor localization algorithms have been extensively studied, and a number of approaches has been proposed over the past two decades [2,3]. These algorithms can be classified into two categories: model-based approaches and fingerprinting-based approaches.

### 2.1. Model-Based Approaches

Model-based approaches use geometrical models, such as time of arrival (ToA) [19] and time difference of arrival (TDoA) [20], to estimate the position of the target [21,22]. Several model-based approaches employing radio propagation models have been investigated in [23]. The log-distance path model, which estimates the RSS value based on the propagation distances, is one of the most popular models. For instance, weighted path loss (WPL) [24] can achieve a 2-m localization accuracy on RFID-based IPS. The AP locations and floor plans are also leveraged to improve the estimation of the RSS value for model-based approaches [7].

However, establishing a sophisticated indoor radio propagation model is very difficult, due to the irregular signal propagation in an indoor environment. Moreover, model-based approaches also suffer from the interference from different materials and mobile obstacles.

### 2.2. Fingerprinting-Based Approaches

A large body of existing IPSs adopt the other type of localization algorithms, namely the fingerprinting-based approaches, which usually involve two phases: an offline training phase and an online localization phase. During the offline training phase, a site survey dedicated to collecting the WiFi RSS fingerprints from different APs at some known locations is performed in the indoor environment, and consequently, a WiFi RSS fingerprint database is built up. During the online localization phase, when a user sends a location query containing his or her current WiFi RSS fingerprint, the location of the user will be estimated by matching the measured fingerprint with the fingerprints stored in the database, and the location associated with the matching fingerprint will be returned as his or her location estimation.

A majority of these approaches leverage RF signals for RSS fingerprinting. To name a few, Horus [8] is based on WiFi signals, while LANDMARC [25] utilizes RFID signals; FM radio [26], geomagnetisms [27,28] and GSM signals [29] are also adopted as fingerprints for indoor localization. Although fingerprinting-based approaches require a site survey to build up a fingerprint database during the offline phase, they can provide a higher localization accuracy than model-based approaches in general [2].

One of the disadvantages of existing fingerprinting-based approaches is the high calibration cost, as a labor-intensive offline site survey is required to build up the RSS fingerprint database. Moreover, the static database is vulnerable to environmental dynamics. The offline site survey needs to be reconstructed when any environmental factor is altered, which involves extensive maintenance costs and efforts.

## 3. An Indoor Localization Algorithm

### 3.1. Preliminary on OS-ELM

ELM is a type of machine learning algorithm based on a single-hidden layer feedforward neural network (SLFN) architecture. OS-ELM on the basis of ELM was developed for SLFNs with additive hidden nodes [15]. Assume there are *N* arbitrary distinct training samples (x*_i_*, t*_i_*) ∈ **R***^n^* × **R***^m^*, where x are the training inputs and **t** are the training targets. If a SLFN with *L* hidden nodes can approximate these *N* samples with zero error, there exist *β_i_*, a*_i_* and *b_i_*, such that:

(1) $$f_{L}\left({\text{x}}_{j}\right)=\sum_{i=1}^{L}\beta_{i}G\left({\text{a}}_{\text{i}},b_{i},{\text{x}}_{j}\right)={\text{t}}_{j},j=1,2,\ldots ,N$$

where a*_i_* and *b_i_* are the learning parameters of the hidden nodes, *β_i_* is the output weight and *G*(*a*_i_, *b_i_*, x*_j_*) is the activation function, which gives the output of the *i*-th hidden node with respect to the input x*_j_*. If the hidden node is additive, *G*(a_i_,*b_i_*,x*_j_*) = *g*(*a*_i_•*x_j_* + *b_i_*), *b_i_* ∈ *R*, where a*_i_* is the input weight vector, *b_i_* is the bias of the *i*-th hidden node and a_i_ • x*_j_* denotes the inner product of the two.

The OS-ELM algorithm contains two phases: an initialization phase and a sequential learning phase. One special property of OS-ELM is that it can learn data one-by-one or chunk-by-chunk (a block of data) with a fixed or varying chunk size [15]. Suppose the network has *L* hidden nodes and the data 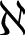 = {(x*_i_*, t*_i_*) |x*_i_* ∈ R*^n^*, t*_i_* ∈ R*^m^*, *i* = 1,…, *N*} are presented to the network sequentially. In the initialization phase, *rank*(**H**_0_) = *L* is required to ensure that OS-ELM can achieve the same learning performance as ELM, where **H**_0_ denotes the hidden output matrix for the initialization phase. The number of training data required in the initialization phase, *N*_0_, has to be equal to or greater than *L, i.e., N*_0_ ≥ *L*. If *N*_0_ = *N*, the performance of OS-ELM is the same as batch ELM. Therefore, batch ELM is a special case of OS-ELM when all of the data are used in the initialization phase.

Initialization phase: a small chunk of training data 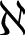
_0_ is used to initialize the learning, where $N_{0}={\left\{{\text{x}}_{i},{\text{t}}_{i}\right\}}_{i=1}^{N_{0}}\subseteq N$ and *N*_0_ ≥ *L*.

Step 1 Randomly assign the input parameters: input weights a*_i_* and bias *b_i_,i*= 1,…, *L*.
Step 2 Calculate the initial hidden layer output matrix **H**_0_ =
  (2) $${\left[\begin{array}{ccc} G\left({\text{a}}_{1},b_{1},{\text{x}}_{1}\right) & \ldots & G\left({\text{a}}_{L},b_{L},{\text{x}}_{1}\right)\\ \vdots & \ldots & \vdots \\ G\left({\text{a}}_{1},b_{1},{\text{x}}_{N_{0}}\right) & \ldots & G\left({\text{a}}_{L},b_{L},{\text{x}}_{N_{0}}\right) \end{array}\right]}_{N_{0}\times L}$$
Step 3 Estimate the initial output weight *β*^(0)^. Since ${\text{T}}_{0}={\left[{\text{t}}_{1},\ldots {\text{t}}_{N_{0}}\right]}_{N_{0}\times m}^{T}$, the problem is equivalent to minimizing ‖**H**_0_*β* − **T**_0_‖. Noticing that **H**† = (**H***^T^***H**)^−1^**H***^T^* [14], the optimal solution is given by $\beta^{\left(0\right)}={\text{P}}_{0}{\text{H}}_{0}^{\text{T}}T_{0}$, where ${\text{P}}_{0}={\left({\text{H}}_{0}^{T}{\text{H}}_{0}\right)}^{-1}$ and ${\text{K}}_{0}-{\text{P}}_{0}^{-1}={\text{H}}_{0}^{T}{\text{H}}_{0}$.
Step 4 Set *k* = 0, where *k* is a parameter indicating the number of chunks of data that are presented to the network.

Sequential learning phase: present the (*k* + 1)-th chunk of new observations ${\text{N}}_{k+1}={\left\{\left({\text{x}}_{i},{\text{t}}_{i}\right)\right\}}_{i=\left(\sum_{j=0}^{k}N_{j}\right)+1}^{\sum_{j=0}^{k+1}N_{j}}$, where *N_k_*_+1_ denotes the number of observations in the (*k* + 1)-th chunk.

Step 1 Compute the partial hidden layer output matrix **H***_k_*_+1_ =
  (3) $${\left[\begin{array}{ccc} G\left({\text{a}}_{1},b_{1},{\text{x}}_{\left(\sum_{j=0}^{k}N_{j}\right)+1}\right) & \ldots & G\left({\text{a}}_{L},b_{L},{\text{x}}_{\left(\sum_{j=0}^{k}N_{j}\right)+1}\right)\\ \vdots & \ldots & \vdots \\ G\left({\text{a}}_{1},b_{1},{\text{x}}_{\sum_{j=0}^{k+1}N_{j}}\right) & \ldots & G\left({\text{a}}_{L},b_{L},{\text{x}}_{\sum_{j=0}^{k+1}N_{j}}\right) \end{array}\right]}_{N_{k+1}\times L}$$
Step 2 Calculate the output weight *β*^(^*^k^*^+1)^. We have ${\text{T}}_{k+1}={\left[{\text{t}}_{\left(\sum_{j=0}^{k}N_{j}\right)+1},\ldots ,{\text{t}}_{\sum_{j=0}^{k+1}N_{j}}\right]}_{N_{k+1}\times m}^{T}$. Moreover,
  (4) $${\text{K}}_{k+1}={\text{K}}_{k}+{\text{H}}_{k+1}^{T}{\text{H}}_{k+1}$$
  (5) $$\beta^{\left(k+1\right)}=\beta^{\left(k\right)}+{\text{K}}_{k+1}^{-1}{\text{H}}_{k+1}^{\text{T}}\left(T_{k+1}-{\text{H}}_{k+1}\beta^{\left(k\right)}\right)$$
  In order to avoid inverting matrices, such as ${\text{K}}_{k+1}^{-1}$ in Equation (5) in the recursive process, the Woodbury formula [30] is applied to transform the equations as follows:
  (6) $${\text{K}}_{k+1}^{-1}={\text{K}}_{k}^{-1}-{\text{K}}_{k}^{-1}{\text{H}}_{k+1}^{T}{\left(\text{I}+{\text{H}}_{k+1}{\text{K}}_{k}^{-1}{\text{H}}_{k+1}^{T}\right)}^{-1}{\text{H}}_{k+1}{\text{K}}_{k}^{-1}$$
  Since ${\text{P}}_{k+1}={\text{K}}_{k+1}^{-1}$,
  (7) $${\text{P}}_{k+1}={\text{P}}_{k}-{\text{P}}_{k}{\text{H}}_{k+1}^{T}{\left(\text{I}+{\text{H}}_{k+1}{\text{P}}_{k}{\text{H}}_{k+1}^{T}\right)}^{-1}{\text{H}}_{k+1}{\text{P}}_{k}$$
  (8) $$\beta^{\left(k+1\right)}=\beta^{\left(k\right)}+{\text{P}}_{k+1}{\text{H}}_{k+1}^{\text{T}}\left(T_{k+1}-{\text{H}}_{k+1}\beta^{\left(k\right)}\right)$$
Step 3 Set *k* = *k* + 1. Go to Step 2 in this online sequential learning phase.

### 3.2. OS-ELM-Based Indoor Localization Algorithm

The proposed OS-ELM approach considers the localization problem as a regression problem. For WiFi RSS fingerprint calibration process, OS-ELM only requires a relative sparse radio map of WiFi RSS during the offline phase, which trumps the traditional fingerprinting-based localization algorithms. These WiFi RSS fingerprints and their physical locations are adopted as training inputs and training targets, respectively, to build up an initial OS-ELM model for online localization.

Furthermore, by leveraging the online learning ability of OS-ELM, WiFi RSS fingerprints can also be collected at some known locations during the online phase to reflect the environmental dynamics. Once new WiFi RSS fingerprints have been collected, they will be integrated into the initial OS-ELM model to update and generate a revised OS-ELM model. During the online phase, when a user sends a location request with his or her current WiFi RSS fingerprint, the fingerprint will be fed into the latest revised OS-ELM model, and then, the estimated location will be calculated.

**Figure 1.**
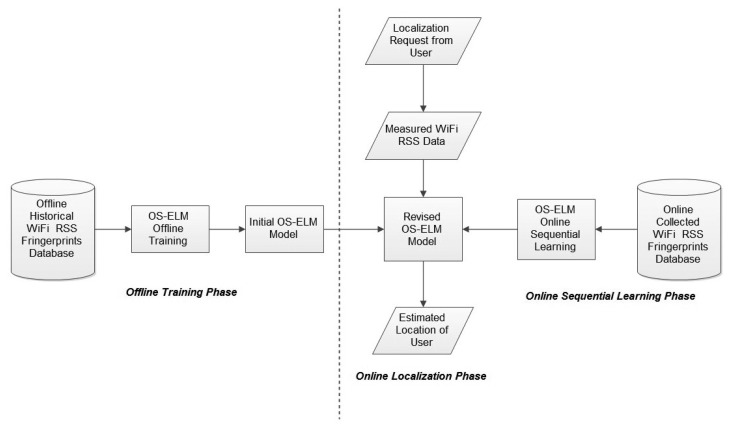
Methodology of the online sequential extreme learning machine (OS-ELM) approach.

The methodology and framework of the proposed OS-ELM approach are demonstrated in Figure 1. The processes of the three main phases of the OS-ELM approach are presented as follow:

#### 3.2.1. Offline Calibration Phase

Suppose 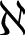
_0_ WiFi RSS fingerprints are collected at some known locations during the offline calibration phase. These WiFi RSS fingerprints and their corresponding physical locations are adopted as the training inputs **x** and the training targets **t**, respectively, for OS-ELM offline training. Similar to the initialization phase of OS-ELM, the initial OS-ELM model will be trained as mentioned in Section 3.1. The detailed steps are illustrated below:

Step 1 Randomly assign the input parameters: input weights a*_i_* and input bias *b_i_*.
Step 2 Calculate the initial hidden layer output matrix **H**_0_.
Step 3 Estimate the initial output weight *β*^(0)^.
Step 4 Set *k* = 0, where *k* indicates the number of updating times of WiFi RSS fingerprints that are collected during the online calibration phase.

Additionally, the activation function *G* and the number of hidden nodes *L* need to be carefully tuned in order to guarantee that the initial OS-ELM model can provide sufficient localization accuracy for the users before any WiFi RSS fingerprints are collected at any online calibration points during the online phase. The five-fold cross-validation method with a range from zero to 1000 and a step size of 50 is employed to select the optimal number of hidden nodes *L*. A guideline for selecting the type of activation functions and the number of hidden nodes is presented in Section 5.

#### 3.2.2. Online Sequential Learning Phase

The main purpose of the online sequential learning phase of OS-ELM is to make the localization algorithm more robust and be able to adapt to various environmental changes. Unlike other online sequential learning algorithms, which can only handle data one-by-one, OS-ELM can learn data one-by-one or chunk-by-chunk with a varying chunk size.

Therefore, WiFi RSS fingerprints can be collected and updated more flexibly for OS-ELM online sequential learning. These newly collected WiFi RSS fingerprints and their corresponding physical locations will be adopted as training samples, and they will be updated chunk-by-chunk to revise the initial OS-ELM model during the online sequential learning phase. Suppose 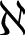
*_k_*_+1_ WiFi RSS fingerprints have been collected during the (*k* + 1)-th online calibration; the revised OS-ELM model will be obtained by the following steps:

Step 1 Calculate the partial hidden layer output matrix *H_k_*_+1_.
Step 2 Calculate the output weight *β*^(^*^k^*^+1)^.
Step 3 Set *k* = *k* + 1 for the next online calibration.

#### 3.2.3. Online Localization Phase

Before any WiFi RSS fingerprints are collected at any known locations during the online phase, the initial OS-ELM model will be used to provide estimated locations of users when they send location queries with their real-time WiFi RSS fingerprints.

When more WiFi RSS fingerprints are collected at some known locations during the online calibration phase, the OS-ELM model is updated and revised. It will be used to provide the estimated location of the user.

## 4. Simulation Results and Evaluation

**Figure 2.**
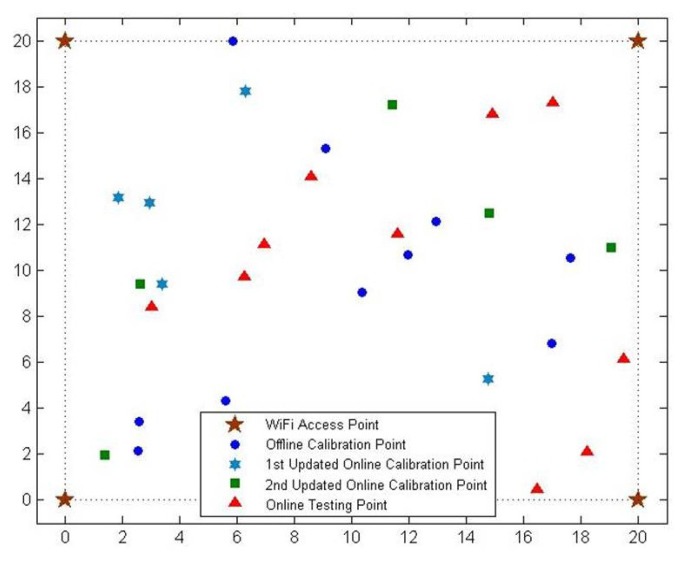
Positions of the WiFi access points, offline calibration points, online calibration points and online testing points in the simulated field.

We develop a simulation environment using MATLAB R2013a in order to evaluate the performance of the proposed OS-ELM approach before any experiment is conducted. The simulation is running on a PC, which has a Intel Core i5-2400 3.10-GHz CPU and 8 GB RAM. As shown in Figure 2, we assume a 20 m × 20 m room, where four WiFi access points are installed at the four corners of the room. The most commonly-used path loss model for indoor environment is the International Telecommunication Union (ITU) indoor propagation model [31]. Since it provides a relation between the total path loss *PL* (dBm) and distance *d* (m), it is adopted to simulate the WiFi signals generated from each WiFi access point. The indoor path loss model can be expressed as:

(9) $$PL\left(d\right)=PL_{0}-10\alpha \text{log}\left(d\right)+X_{\sigma }$$

where *PL*_0_ is the pass loss coefficient, and it is set to be −40 dBm in our simulation. *X_σ_* represents a zero-mean normal random noise with standard deviation *σ* = 0.5, and *α* is the path loss exponent.

During the offline calibration phase, *α* is set to be two in Scenario I, and simulated WiFi RSS fingerprints from the four WiFi routers are collected at 10 randomly selected offline calibration points for OS-ELM offline training, with 200 WiFi RSS fingerprints collected at each point. The hardlim function *G*(*a, b, x*) = *hardlim*(*ax* + *b*) is chosen as the activation function, and 380 hidden nodes are selected and put in the hidden layer during the offline training. The initial OS-ELM model is obtained after a 0.219-s training process. In order to imitate the environmental dynamics in the room, we set *α* to be 2.5 in Scenario II and 3.5 in Scenario III, respectively. WiFi RSS fingerprints are collected at five online calibration points and five online testing points under each scenario. Two hundred WiFi RSS fingerprints are collected at each point, and the positions of these points are distinct. The positions of the offline calibration points, the online calibration points and the online testing points are demonstrated in Figure 2. The updated WiFi RSS fingerprints at the online calibration points are adopted as online training samples and leveraged to revise the initial OS-ELM model.

We evaluate the performance of OS-ELM with each online sequential learning updated based on the WiFi RSS fingerprints collected at the 10 online testing points. The distance error is used to measure the localization accuracy of the proposed OS-ELM approach. We define the location estimation error *e* to be the distance between the real location coordinates (*x*_0_, *y*_0_) and the system estimated location coordinates (*x,y*), *i.e.*,:

(10) $$e=\sqrt{{\left(x-x_{0}\right)}^{2}+{\left(y-y_{0}\right)}^{2}}$$

**Figure 3.**
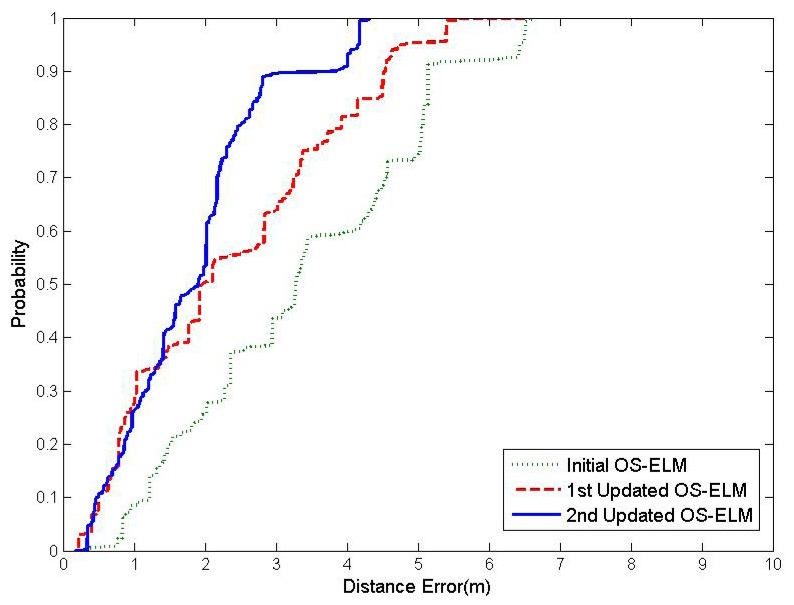
Cumulative percentile of error distance.

Table 1 illustrates the performance of OS-ELM with each online sequential learning update in terms of training time, testing time and average localization accuracy. The comparison of the cumulative percentile of error distances between the initial OS-ELM and the two updated OS-ELM is presented in Figure 3. It can be easily spotted from Table 1 that the average localization accuracy of OS-ELM becomes better when more WiFi RSS fingerprints at various online calibration points have been learned online. The latest updated OS-ELM can provide 1.794 m, which enhances the precision of indoor localization by 42.18% over the performance of the initial OS-ELM. Furthermore, the online sequential learning of OS-ELM is very efficient. It only spends 0.014 s on average to calculate the output weights *β* for 1000 newly-received WiFi RSS fingerprints in each online sequential learning update.

Based on the simulation results and evaluation, we can conclude that OS-ELM can provide higher localization accuracy due to its efficient online sequential learning ability when the indoor environment is altered during the online phase.

## 5. Experimental Results and Performance Evaluation

### 5.1. System Overview

We conducted extensive experiments to evaluate the performance of the proposed OS-ELM approach. The testbed is Internet of Things Laboratory at School of Electrical and Electronic Engineering, Nanyang Technological University. The area of the test-bed is around 580 m^2^ (35.1 m × 16.6 m). Thirty six graduate students and 15 undergraduate students work and study in this lab regularly.

**Figure 4.**
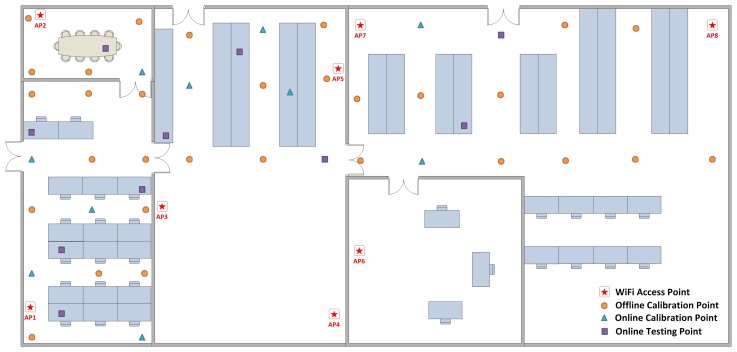
Positions of the WiFi access points, offline calibration points, online calibration points and online testing points in the test-bed.

**Figure 5.**
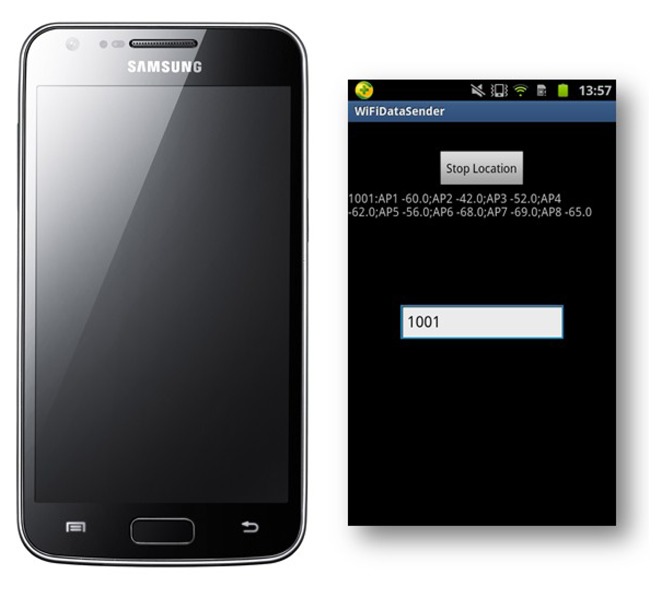
The test mobile device Samsung I929 and developed Android apps.

The layout of the testbed is shown in Figure 4. Eight D-Link DIR-605L WiFi Cloud Routers are utilized as WiFi access points for our experiments. In order to collect WiFi RSS fingerprints from multiple access points simultaneously, an Android application that can collect data once per second is developed. As shown in Figure 5, this Android app is installed on a Samsung I929 Galaxy SII mobile phone. We leveraged this mobile phone to collect the WiFi RSS fingerprints at the offline calibration points, online calibration points and online testing points. After that, all of this information was sent to a process server running on a PC. The performance evaluation was conducted on the server side.

The initial OS-ELM model was built by the following steps. During the offline phase, 30 offline calibration points were selected, and 1000 WiFi RSS fingerprints were collected at each point. The positions of these 30 offline calibration points are demonstrated in Figure 4. The grid spacing between two adjacent locations of the calibration points was chosen to be larger than 1.25 m based on the analysis in [32]. By leveraging these 30,000 WiFi RSS fingerprints and their physical positions as training inputs and training targets accordingly, the initial OS-ELM model was constructed. During the online phase, we continued to collect WiFi RSS fingerprints at several online calibration points and online testing points for five days. In each day, two distinct online calibration points and two distinct online testing points were selected in order to reflect the environmental dynamics. The positions of these total 10 online calibration points and 10 online testing points are also presented in Figure 4. One thousand WiFi RSS fingerprints are collected at each point. Unlike [18], which only tested the performance of OS-ELM in the corridors in the testbed, as shown in Figure 4, the positions of the online calibration points and online testing points were selected on the tables and cubicles, as well as in the testbed to acquire a more comprehensive performance evaluation of the proposed OS-ELM approach.

### 5.2. Selection of Parameters for the Initial OS-ELM Model

Based on the analysis in Section 3.2, the type of activation function and the number of hidden nodes in the OS-ELM hidden layer are two key parameters affecting the performance of OS-ELM during the initialization phase, which is accordingly the offline calibration phase in our case.

#### 5.2.1. Selection of the Type of Activation Function *G* for the Initial OS-ELM Model

By using the 30,000 WiFi RSS fingerprints we collected during the offline calibration phase, we evaluate the performance of three different activation functions: radial basis function (RBF) *G*(*a, b, x*) = *e^−b^*^‖^*^x−a^*^‖2^, sine function *G*(*a, b, x*) = *sin*(*ax*+*b*) and hard-limit transfer (hardlim) function *G*(*a, b,x*) = *hardlim*(*ax* + *b*), with different numbers of hidden nodes.

**Figure 6.**
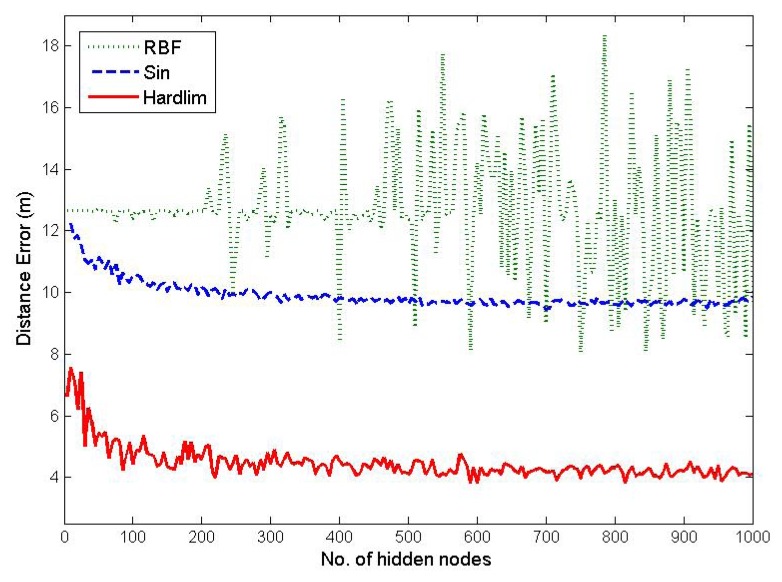
Localization accuracy regarding different activation functions and different numbers of hidden nodes.

It can be seen from Figure 6 that as the number of hidden nodes increases, the performances in terms of the mean localization accuracy of the sine function and hardlim function become better. On the contrary, the performance of RBF is the worst and appears to be irrelevant with respect to the number of hidden nodes. Since the performance of the hardlim function is the best among the three activation functions, it is chosen as the activation function for the initial OS-ELM model and also for the online sequential learning of OS-ELM.

#### 5.2.2. Selection of the Number of Hidden Nodes *L* for the Initial OS-ELM Model

Another critical parameter for the performance of the OS-ELM approach is the number of hidden nodes *L* in the initial OS-ELM hidden layer, after the hardlim function is chosen as the activation function. The five-fold cross-validation method is employed with a range from 0 to 1000 and a step size of 50 in order to determine the optimal *L*. As observed in Figure 6, the performances in terms of the localization accuracy of the hardlim function become relatively stable when *L* is increased to 600.

In addition to the evaluation of the localization accuracy, the repeatability (REP) of the initial OS-ELM with a different number of hidden nodes is also evaluated by leveraging the 30,000 WiFi RSS fingerprints that we collected during the offline calibration phase. REP is measured by the standard deviation of localization errors over the *r* repeated realizations, and this measure is proposed based on the fact that ELM with the same parameters and in the same training dataset may draw quite different results. A smaller value of REP is desired in general. The mean localization error *ē* and REP are calculated based on the following equations:

(11) $$\bar{e}=\frac{1}{r}\sum_{m=1}^{r}e_{m}$$

(12) $$\text{REP}=\sqrt{\frac{1}{r}\sum_{m=1}^{r}\left(e_{m}-\bar{e}\right)}$$

**Figure 7.**
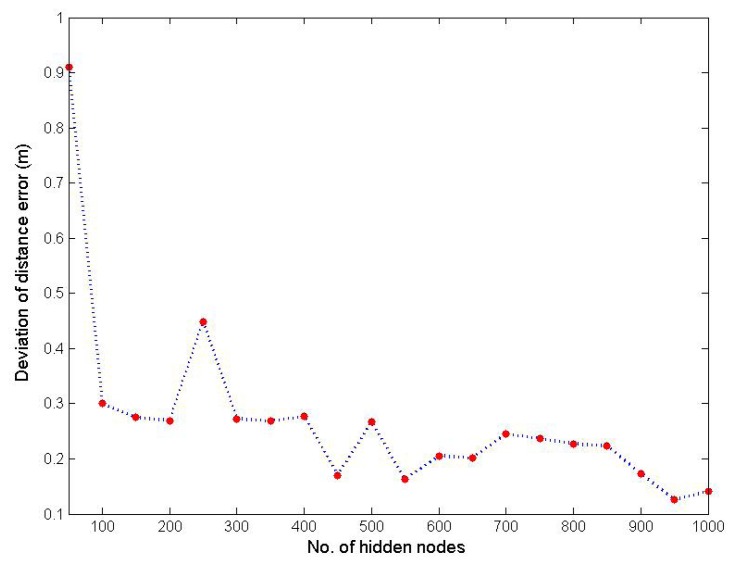
Deviation of distance error with different numbers of hidden nodes.

*r* in our experiment is selected as 50. Figure 7 demonstrates the repeatability of the initial OS-ELM, which is measured by the standard deviation of localization error with a range of hidden nodes from zero to 1000 and a step size of 50. As observed from Figure 7, the standard deviation of localization errors decreases when the number of hidden nodes is increased. After comprehensive evaluations on both localization accuracy and repeatability, *L* is selected to be 950.

A guideline for selecting the type of activation function and the number of hidden nodes in the OS-ELM hidden layer, both of which are the critical parameters for the performance of the OS-ELM approach, is listed as follows: the suggested default activation function for the OS-ELM approach is the hardlim function, whose performance is better than others generally in terms of simulation and experimental results. As for the optimal number of hidden nodes, the five-fold cross-validation method is employed with a range from 0 to 1000 and a step size of 50 based on the empirical tuning.

### 5.3. Comparison between OS-ELM and Other Methods

Three well-known algorithms, RADAR K nearest neighbor (KNN) [7], fuzzy K nearest neighbor (Fuzzy KNN) [33] and batch ELM [14], are chosen to compare with the proposed OS-ELM approach. It has been shown in [34,35] that the performance of batch ELM in terms of the offline training time, the online testing time and the average localization accuracy is better than classical machine learning algorithms, such as the back-propagation (BP) algorithm and the support vector machine for regression (SVR) algorithm. Therefore, we choose batch ELM to be compared with the proposed OS-ELM. Considering the wide usage of KNN and fuzzy KNN as classical localization algorithms, we also include them in the comparison.

Unlike OS-ELM, which can update and revise the initial OS-ELM model sequentially during the online phase, KNN, fuzzy KNN and batch ELM can only utilize the data collected during the offline phase. In order to make a fair comparison, we collected WiFi RSS fingerprints, not only at the 30 offline calibration points, but also at the 10 online calibration points, to build up the WiFi RSS fingerprints offline database during the offline calibration phase. One thousand WiFi RSS fingerprints were collected at each point. The hardlim function and 950 hidden nodes in the hidden layer were chosen for batch ELM offline training, which are the same as for the initial OS-ELM offline training. The batch ELM model was obtained by leveraging the WiFi RSS fingerprints and their corresponding locations stored in the database. During the online phase, after feeding the WiFi RSS fingerprints into the batch ELM model, this model will output the estimated location of the target. For KNN and fuzzy KNN, the value of *K* was chosen empirically based on the data stored in the database. During the online phase, by matching the measured WiFi RSS fingerprints with the *K* closest WiFi RSS fingerprints in the database, the location of the target will be calculated. The detail methodologies of these are presented in [7,33].

The comparison of performances will be conducted as follows. First of all, we evaluate the performances of KNN, fuzzy KNN, batch ELM and OS-ELM without online sequential learning based on the WiFi RSS fingerprints we collected at the 10 online testing points during the online localization phase. Table 2 demonstrates the performance comparison between KNN, fuzzy KNN, batch ELM and OS-ELM in terms of the training time, the testing time and the average localization accuracy. As shown in Table 2, although the localization accuracy of the initial OS-ELM is slightly worse than that of batch ELM, the offline training time of OS-ELM is less than that of batch ELM by 30.01%, which evidently reduces the time and manpower costs for the offline site survey. The testing times of KNN, fuzzy KNN, batch ELM and OS-ELM are almost the same.

During the online phase, since we collected WiFi RSS fingerprints at two different online calibration points at each time, the performance of OS-ELM with each online sequential learning update is also presented in Table 2. As observed from Table 2, the average localization accuracy of OS-ELM becomes better when more WiFi RSS fingerprints at different online calibration points have been learned online. In addition, another noteworthy point is that the online sequential learning time of OS-ELM is quite fast. It only spends 1.2108 s on average to calculate the output weights *β* for 2000 newly-received WiFi RSS fingerprints in each online sequential learning update.

**Figure 8.**
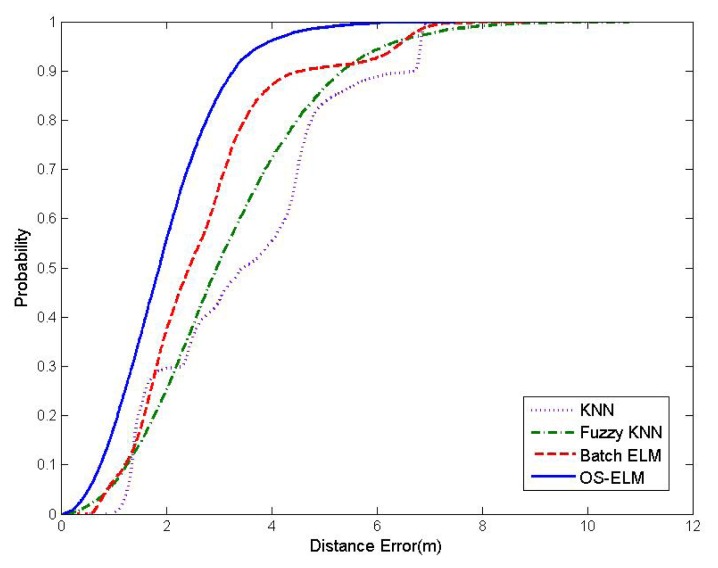
Cumulative percentile of error distance for different methods.

**Figure 9.**
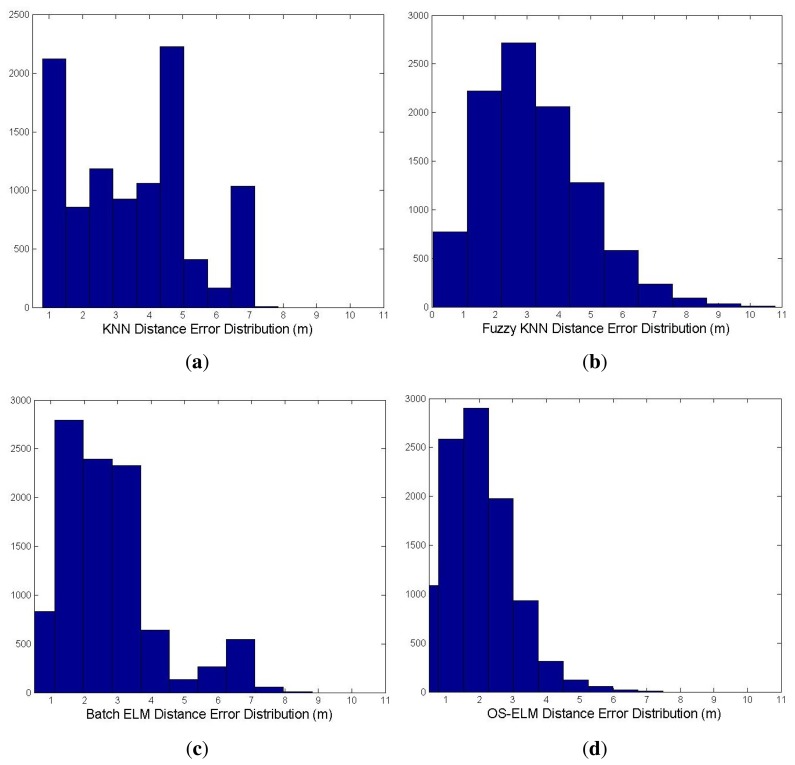
Comparison of the distance error distribution for different methods. (**a**) KNN; (**b**) fuzzy KNN; (**c**) batch ELM; (**d**) OS-ELM.

As observed from Table 2, the average localization accuracy by using KNN, fuzzy KNN and batch ELM is, respectively, 3.098 m, 2.728 m and 2.581 m. In contrast, with online sequential learning of WiFi RSS fingerprints at 10 different online calibration points, OS-ELM can provide a localization accuracy of 1.973 m, which enhances the precision of indoor localization by 36.31%, 27.68% and 23.56% over KNN, fuzzy KNN and batch ELM, respectively. The comparison of the cumulative percentile of error distances between KNN, fuzzy KNN, batch ELM and the latest updated OS-ELM is presented in Figure 8. Figure 9 demonstrates the distance error distribution of the four approaches. The distance error distribution of OS-ELM, as shown in Figure 9d, ranges mainly within 2.5 m. On the contrary, the distance error distributions of KNN in Figure 9a, fuzzy KNN in Figure 9b and batch ELM in Figure 9c are much more scattered.

In summary, based on our experimental results and analysis, we can conclude that OS-ELM can provide higher localization accuracy consistently with a fast online sequential learning speed to adjust to various environmental dynamics than existing approaches, such as KNN, fuzzy KNN and batch ELM.

### 5.4. Performance Evaluation of OS-ELM under Specific Environmental Dynamics

Variations of occupancy distribution and events of opening and closing of doors have been observed as two major environmental factors that could severely affect the localization accuracy of WiFi-based IPS, since they change frequently over time in the indoor environment [6]. Besides the general performance evaluation of the proposed OS-ELM approach, as shown in [18] and the above sections, we also conducted experiments to evaluate how well OS-ELM adapts to some specific environmental dynamics. During each experiment, we measured the localization accuracy of the system when only one environmental factor was altered, while others remained unvaried.

#### 5.4.1. Impact of Human Presence and Movements

This experiment aims to determine how well the proposed OS-ELM approach can adjust to the interferences of human presence and movements while other environmental factors are kept unchanged. The experiment was conducted in the conference room in the testbed, since it is easier to create and manage the variation of the occupancy distribution for performance evaluation in this relatively small space. As shown in Figure 1, the location of the conference room is the left upper corner of the testbed. The area of the conference room is around 22 m^2^ (6.3 m × 3.5 m).

The experiment was conducted based on the following steps. First of all, we continued to collect WiFi RSS fingerprints at five offline calibration points for one day when nobody was in the conference room. After that, the batch ELM model and the initial OS-ELM model were built up based on the collected WiFi RSS fingerprints and their corresponding positions. We put the testing mobile device on the conference table to evaluate the performances of the batch ELM and the OS-ELM under the non-human interference condition in the first place. As shown in Table 3, the batch ELM and the OS-ELM provide similar localization accuracy under the non-human interference condition, which is 2.203 m and 2.183 m, respectively.

Then, we evaluate the performance of both batch ELM and OS-ELM with human interference. The position of the online testing point was unchanged. However, five people intentionally sat around the table for 6 h. The purpose is to use human bodies to disturb or block the WiFi signal transmission between the test mobile device and the multiple WiFi access points. Meanwhile, WiFi RSS fingerprints were collected at three online calibration points to update and revise the OS-ELM model.

After conducting the experiment for 6 h, the average localization accuracies of the two approaches are calculated and demonstrated in Table 3. It can be seen from Table 3 that the performance of batch ELM becomes worse with human interference. Its performance decay is 41.99% compared with non-human interference. The main reason is that the batch ELM model was constructed under non-human interference, which cannot reflect the variation of occupancy distribution adaptively during the online phase. On the contrary, by leveraging the information collected at the online calibration points, OS-ELM can nicely capture the event of human presence and movements and take this environmental change into the online localization process sequentially. As shown in Table 3, the average localization accuracy provided by OS-ELM under the human interference circumstance is 2.197 m, almost the same as the non-human interference scenario. It enhances the precision of indoor localization by 29.27% over batch ELM under the same condition.

To conclude, OS-ELM can provide high localization accuracy consistently when the occupancy distribution is altered due to its fast adaptation.

#### 5.4.2. Impact of Opening/Closing of Doors

Besides the variation of occupancy distribution, another environmental factor that affects the performance of WiFi-based IPS badly is the events of opening and closing of doors. Therefore, we also conducted an experiment to evaluate how well the proposed OS-ELM approach can adjust to opening and closing of doors while other environmental factors were kept unchanged.

The experiment was conducted based on the following steps. We collected WiFi RSS fingerprints at 15 offline calibration points, which were near the four doors inside the testbed for one day under the all-door-open condition firstly. The batch ELM model and the initial OS-ELM model were built up by leveraging the collected WiFi RSS fingerprints and their corresponding positions. Then, we determined the performance of these two approaches near the door area. It can be seen from Table 4 that both batch ELM and OS-ELM can provide nearly 2.15-m localization accuracy on average when all doors are opened.

After that, we continued the experiment to assess the performance of these two approaches under the all-door-close condition. We closed the four doors within the testbed for 6 h and collected WiFi RSS fingerprints at the same online testing points as under the all-door-open condition. WiFi RSS fingerprints were also collected at the 10 online calibration points in the testbed simultaneously for updating the OS-ELM model.

The average localization accuracy of batch ELM and OS-ELM under the all-door-close condition is presented in Table 4. As shown in Table 4, the average localization accuracy of batch ELM is 4.203 m when all doors are closed, which is 47.32% worse than the all-door-open condition. This reveals that batch ELM is not capable of providing a sufficient and satisfactory indoor positioning service when the status of doors is different from the offline calibration phase.

In contrast, the performance of OS-ELM is more stable and robust when the status of doors is altered. It can be seen in Table 4 that the average localization accuracy provided by OS-ELM under the all-door-close condition is 2.085 m, which is roughly the same as the all-door-open condition. Moreover, it enhances the precision of indoor localization by 50.39% over batch ELM. The main reason that OS-ELM can consistently provide the indoor positioning service under different status of doors is due to its online sequential learning ability. By leveraging the information collected at the online calibration points, OS-ELM can sequentially update its model to adjust the influence from opening or closing doors.

Thus, the experimental results leads to the same conclusion that OS-ELM can provide higher localization accuracy steadily under various environmental dynamics, regardless of the impact from human presence or movements or the influence from changing the status of doors.

## 6. Conclusions and Future Work

In this paper, we proposed an indoor localization algorithm based on OS-ELM to address the two challenging problems of the existing WiFi-based IPS: the intensive costs of manpower and time for offline site survey and the inflexibility to environmental dynamics. Both our simulation analysis and experimental studies have shown that OS-ELM can tackle the problems satisfactorily. The fast learning speed of OS-ELM obviously reduced the time consumptions and manpower costs for the site survey during the offline calibration phase. In addition, the online sequential learning ability of OS-ELM made it possible to reflect and adapt to the environmental changes in a timely manner. Furthermore, WiFi RSS fingerprints can be collected and updated more flexibly, since OS-ELM can learn data with a varying chunk size. Experiments under specific environmental dynamics, such as variations of the occupancy distribution and events of opening or closing doors, were also conducted. In summary, OS-ELM can provide high localization accuracy with a fast online sequential learning speed under various environmental changes and achieve superior performance to the existing approaches.

Future work can be focused on the following directions. Firstly, since we only conducted experiments in the lab environment, experiments in other indoor scenarios, such as lecture theaters or shopping centers, need to be conducted to make the performance evaluation of OS-ELM more conclusive. Secondly, mobile devices with different brands or equipped with different WiFi chipsets as the one used in the experiments will incur degraded localization accuracy. Therefore, how to improve OS-ELM to overcome the heterogeneous issue among mobile devices is quite attractive.

## Figures and Tables

**Table 1.** Simulation results of OS-ELM.

**Number of Calibration Points (Offline + Online)**	**Training Time (s)**	**Testing Time (s)**	**Accuracy (m)**
10 + 0	0.219	0.015	3.103
10 + 5	0.148	0.014	2.563
15 + 5	0.139	0.014	**1.794**

**Table 2.** Comparison between OS-ELM and other methods.

**Approaches**	**Number of Calibration Points** (**Offline + Online**)	**Training Time** (**s**)	**Testing Time** (**s**)	**Accuracy** (**m**)
**KNN**	40 + 0	-	0.108	3.098

**Fuzzy KNN**	40 + 0	-	0.117	2.728

**Batch ELM**	40 + 0	11.574	0.113	2.581

**OS-ELM**	30 + 0	8.101	0.113	2.615
	30 + 2	1.204	0.111	2.487
	32 + 2	1.213	0.114	2.346
	34 + 2	1.182	0.109	2.219
	36 + 2	1.226	0.115	2.091
	38 + 2	1.229	0.117	**1.973**

**Table 3.** Impact of human presence and movements on localization accuracy.

**Localization Accuracy (m)**	**Non-Human Interference**	**With Human Interference**
Batch ELM	2.203	3.106
OS-ELM	2.183	**2.197**

**Table 4.** Impact of opening/closing doors on localization accuracy.

**Localization Accuracy (m)**	**All Doors are Opened**	**All Doors are Closed**
Batch ELM	2.214	4.203
OS-ELM	2.107	**2.085**
